# Impact of Carcinomatosis on Clinical Outcomes after Self-Expandable Metallic Stent Placement for Malignant Gastric Outlet Obstruction

**DOI:** 10.1371/journal.pone.0140648

**Published:** 2015-10-14

**Authors:** Ji Eun Lee, Keol Lee, Yun Soo Hong, Eun Ran Kim, Hyuk Lee, Byung-Hoon Min

**Affiliations:** Department of Medicine, Samsung Medical Center, Sungkyunkwan University School of Medicine, Seoul, Korea; Hokkaido University, JAPAN

## Abstract

**Background:**

It is still unclear whether the peritoneal carcinomatosis had a negative effect on the clinical outcomes of patients who underwent self-expandable metallic stent (SEMS) placement for malignant gastric outlet obstruction (GOO). Although carcinomatosis may be associated with the development of multifocal gastrointestinal (GI) tract obstruction or decreased bowel movement, previous studies investigated the occurrence of stent failure only and thus had limitation in evaluating clinical outcomes of patients with carcinomatosis.

**Methods:**

Between 2009 and 2013, 155 patients (88 patients without carcinomatosis and 67 patients with carcinomatosis) underwent endoscopic SEMS placement for malignant GOO. Factors affecting clinical success and obstructive symptom-free survival (time period between SEMS placement and the recurrence of obstructive symptoms due to multifocal GI tract obstruction or decreased bowel movement as well as stent failure) were assessed.

**Results:**

Patients with carcinomatosis showed higher Eastern Cooperative Oncology Group (ECOG) scale than those without carcinomatosis. Clinical success rates were 88.1% in patients with carcinomatosis and 97.7% in patients without carcinomatosis. In multivariate analysis, only ECOG scale was identified as an independent predictor of clinical success. During follow-up period, patients with carcinomatosis showed significantly shorter obstructive symptom-free survival than those without carcinomatosis. In multivariate analysis, the presence of carcinomatosis, chemotherapy or radiation therapy after SEMS placement, and obstruction site were identified as independent predictors of obstructive symptom-free survival. For patient without carcinomatosis, stent failure accounted for the recurrence of obstructive symptoms in 84.6% of cases. For patients with carcinomatosis, multifocal GI tract obstruction or decreased bowel movement accounted for 37.9% of cases with obstructive symptom recurrence and stent failure accounted for 44.8% of cases.

**Conclusions:**

Carcinomatosis predicts unfavorable long-term clinical outcomes in patients undergoing SEMS placement for malignant GOO. This is mainly due to the development of multifocal GI tract obstructions or decreased bowel movement as well as stent failure.

## Introduction

Malignant gastric outlet obstruction (GOO) is defined as the inability of the stomach to empty due to mechanical obstruction by any malignancies at the level of either the distal stomach or the duodenum. This condition is a late complication of advanced gastrointestinal (GI) cancers including gastric, pancreatic and periampullary cancers [1]. Malignant GOO can lead to significant morbidity such as persistent nausea, vomiting, abdominal pain, weight loss, and cachexia. Endoscopic placement of a self-expandable metallic stent (SEMS) has been reported as an effective and safe treatment modality to relieve these symptoms. Currently, SEMS placement is widely used in patients with malignant GOO instead of surgical bypass [1–4].

Peritoneal carcinomatosis is considered a relative contraindication to SEMS placement for malignant GOO given the theoretical risk of multifocal GI tract obstructions and decreased bowel movement [1, 2, 5]. To date, only a few studies have assessed the impact of carcinomatosis on clinical outcomes after SEMS placement for malignant GOO, and the results are conflicting. In a Western study [6], clinical success rates immediately after SEMS placement were comparable between patients with and without carcinomatosis. In two Korean studies [7, 8], however, patients with carcinomatosis showed significantly lower clinical success rates than those without carcinomatosis.

Both stent failure and multifocal GI tract obstruction or decreased bowel movement can affect long-term clinical outcomes after SEMS placement in patients with carcinomatosis. Therefore, both should be assessed to fully evaluate the long-term effect of SEMS placement for malignant GOO in patients with carcinomatosis. However, previous studies investigated the occurrence of stent failure only and did not assess the development of multifocal GI tract obstruction or decreased bowel movement after SEMS placement [6–8]. Thus, they had significant limitation in evaluating clinical outcomes after SEMS placement in patients with carcinomatosis [6–8].

In the present study, we investigated the impact of carcinomatosis on the clinical success and long-term outcomes of patients undergoing SEMS placement for malignant GOO. To overcome the limitation of previous studies, we assessed the occurrence of multifocal GI tract obstruction or decreased bowel movement as well as stent failure after SEMS placement.

## Patients and Methods

### Patients

We performed a retrospective review of patients with malignant GOO who underwent their first endoscopic SEMS placement between January 2009 and February 2013 at Samsung Medical Center. In our institution, stent insertion is the first option for patients with malignant GOO who were not candidates for curative surgery. Surgical bypass is performed only when unexpected distant metastasis or unresectable condition is found during surgery. The present study enrolled patients with any malignancies causing luminal obstruction in distal stomach or duodenum and presenting with symptoms of GOO. None of these patients were candidates for curative surgery due to advanced cancer stage or distant metastasis. Patients were excluded if they had undergone surgical bypass for GOO or had previously received gastric, periampullary, or duodenal surgery for other causes. In addition, patients were excluded if radiologic study revealed evidence of multifocal GI tract obstructions before SEMS insertion. The diagnosis of GOO was made based on upper endoscopy findings or radiologic study including abdominal computed tomography (CT) or upper GI barium study. All enrolled patients had symptoms associated with GOO. The diagnosis of carcinomatosis was made based on CT findings such as ascites, hypovascular omental masses, nodular thickening or enhancement of peritoneum, and soft tissue infiltration into the mesentery [6]. In cases of suspicious carcinomatosis, diagnostic laparoscopy was performed to confirm the presence of carcinomatosis and to determine the treatment plan. All enrolled patients provided written informed consent before undergoing SEMS placement. The study protocol was approved by the institutional review board at Samsung Medical Center (IRB approved number: 2014-09-123).

### SEMS placement

SEMS placement was performed with a therapeutic gastroscope (GIF-2T240, Olympus, Tokyo, Japan) or colonoscope (CF-H260AI, Olympus, Tokyo, Japan) using a through-the-scope method. All patients underwent procedures under a conscious sedation with midazolam and pethidine. Once the area of stenosis was reached, the stricture length was estimated by advancing the endoscope through the stricture site, if possible, or by injection of contrast material with the use of fluoroscopy. The stent length was chosen to ensure at least a 2-cm length of stent to be flared at both ends of the stricture. A guidewire was advanced under fluoroscopic guidance through the stricture, and the SEMS delivery system was placed over the guidewire. The stent was then deployed at the stricture site under endoscopic and fluoroscopic guidance. All SEMSs used were either Bona (Standard Sci Tech, Seoul, Korea) stent or Hanaro stent (M.I.Tech, Seoul, Korea). If SEMS insertion was technically successful without any immediate complications, the patient was allowed clear water intake on the day of the procedure. The diet level was increased gradually to low residual diet if the patient tolerated the diet and the expansion of SEMS was confirmed by simple abdominal radiography.

### Definitions

Technical success was defined as successful deployment of SEMS in the proper position across the stricture site and a confirmation of patency by using a combination of upper endoscopy and fluoroscopy. The degree of oral intake was assessed before and three days after SEMS placement using the GOO scoring system (GOOSS): 0, no oral intake; 1, exclusively liquid diet; 2, exclusively soft solid diet; and 3, low-residue or full diet [9]. Clinical success was defined as relief of the obstructive symptoms and improvement of at least one point in the GOOSS score at 72 hours after SEMS placement.

Obstructive symptom-free survival was defined as the time period between SEMS placement and the recurrence of obstructive symptoms with consequent decrease in the GOOSS score. If the patient remained free of obstructive symptoms during the follow-up period, obstructive symptom-free survival was measured from the date of SEMS placement to the date of the last follow-up. The causes of recurrence of obstructive symptoms included multifocal GI tract obstruction or decreased bowel movement as well as stent failure. The diagnosis of multifocal GI tract obstruction was made based on the radiologic study including abdominal CT or upper GI barium study. Stent failure included stenosis by tumor ingrowth or overgrowth, migration, collapse, and fracture. If there were no evidences of multifocal GI tract obstruction or stent failure and patients showed decreased or no bowel sound in auscultation, patients were diagnosed to have decreased bowel movement.

Performance status was assessed with the Eastern Cooperative Oncology Group (ECOG) scale: 0, normal activity; 1, symptomatic but ambulatory; 2, in bed 50% or less of the time; 3, in bed more than 50% of time; and 4, totally bedridden [8].

### Statistical analysis

Categorical data were analyzed with the Chi-square test or Fisher’s exact test, and continuous data were analyzed with the Student’s *t-*test or the Mann-Whitney *U* test. To compare median values, we used the Wilcoxon rank sum test. Logistic regression analysis was used to identify independent predictors of clinical success assessed three days after SEMS placement. The Kaplan-Meier method was used to evaluate stent patency and obstructive symptom-free survival. Stent patency and obstructive symptom-free survival were assessed in patients who had clinical success of SEMS placement and underwent follow-up. Patients who died without any follow-up examination after SEMS placement were regarded as a follow-up loss and not included in the analysis. Patients who died without recurrent obstructive symptom at the last follow-up were censored at the date of the last follow-up. Censoring date for stent patency and obstructive symptom-free survival was February 28, 2014. Multivariate analysis using the Cox proportional-hazards model was performed to explore the potential association between obstructive symptom-free survival and clinicopathologic parameters (age, gender, ECOG scale, obstruction site, type of SEMS, treatment after SEMS placement including chemotherapy and radiation therapy, and presence of carcinomatosis). A *P-*value less than 0.05 was considered statistically significant.

## Results

### Baseline characteristics of patients according to the presence of carcinomatosis

During the study period, a total of 155 patients (88 patients without carcinomatosis and 67 patients with carcinomatosis) underwent their first endoscopic SEMS placement for malignant GOO. Surgical bypass was performed in 48 patients with malignant GOO in the same period. Table 1 summarizes and compares the baseline characteristics of 155 patients with and without carcinomatosis. Gastric cancer and pancreatic cancer were the most common underlying malignancies in both groups. The carcinomatosis group had significantly higher ECOG scale (poor performance status) than the no carcinomatosis group. In patients with extrinsic compression, mucosal lesion was not observed in GOO site in upper endoscopy. Extrinsic compression accounted for 10.2% and 9.0% of the no carcinomatosis and carcinomatosis group, respectively. In the present study, Bona stent was used in 127 cases (112 uncovered stents and 15 partially covered stents) and Hanaro stent was used in 28 cases (28 uncovered stents).

**Table 1:** Comparison of baseline characteristics according to the presence of carcinomatosis.

	No carcinomatosis (n = 88)	Carcinomatosis (n = 67)	*P* value
Age (yrs)			0.130
Mean ± SD	64.4 ± 12.8	61.2 ± 12.7	
Median (range)	65 (26–88)	61 (37–90)	
Gender (%)			0.291
Male	61 (69.3)	41 (61.2)	
Female	27 (30.7)	26 (38.8)	
ECOG scale			0.033
Mean ± SD	1.8 ± 0.8	2.1 ± 0.9	
Median (range)	2 (1–4)	2 (1–4)	
Underlying malignancy (%)			0.517
Gastric cancer	50 (56.8)	42 (62.7)	
Pancreatic cancer	20 (22.7)	11 (16.4)	
Duodenal cancer	4 (4.5)	0 (0.0)	
AOV cancer	2 (2.3)	1 (1.5)	
GB cancer	3 (3.4)	3 (4.5)	
CBD cancer	3 (3.4)	5 (7.5)	
Esophageal cancer	1 (1.1)	0 (0.0)	
Others	5 (5.7)	5 (7.5)	
Obstruction site (%)			0.250
Antrum/Pyloric ring	52 (59.1)	44 (65.7)	
Duodenal bulb/second portion	30 (34.1)	15 (22.4)	
Duodenal third/fourth portion	6 (6.8)	8 (11.9)	
Type of SEMS (%)			0.780
Uncovered	80 (90.9)	60 (89.6)	
Partially covered	8 (9.1)	7 (10.4)	
Length of SEMS (cm, %)			0.310
< 10	44 (50.0)	28 (41.8)	
≥ 10	44 (50.0)	39 (58.2)	
Extrinsic compression (%)			0.791
No	79 (89.8)	61 (91.0)	
Yes	9 (10.2)	6 (9.0)	

ECOG, Eastern Cooperative Oncology Group; AOV, ampulla of Vater; GB, gall bladder; CBD, common bile duct; SEMS, self-expandable metallic stent

### Technical and clinical success of SEMS placement according to the presence of carcinomatosis

Table 2 demonstrates the technical and clinical success rates of SEMS placement in patients with and without carcinomatosis. Technical success rates were 100% in both groups. The no carcinomatosis group showed significantly higher clinical success rate than the carcinomatosis group in univariate analysis (97.7% versus 88.1%, *P* = 0.020). The median GOOSS scores after SEMS placement were 2 in both groups.

**Table 2:** Technical and clinical success rates of self-expandable metallic stent placement according to the presence of carcinomatosis.

	No carcinomatosis (n = 88)	Carcinomatosis (n = 67)	*P* value
Technical success (%)			1.000
No	0 (0.0)	0 (0.0)	
Yes	88 (100.0)	67 (100.0)	
Clinical success (%)			0.020
No	2 (2.3)	8 (11.9)	
Yes	86 (97.7)	59 (88.1)	
GOOSS score (median (range))			
Before SEMS placement	1 (0–2)	1 (0–1)	0.620
After SEMS placement	2 (0–3)	2 (0–3)	0.977

SEMS, self-expandable metallic stent; GOOSS, gastric outlet obstruction scoring system

In multivariate analysis (Table 3), however, the presence of carcinomatosis was not an independent predictor of clinical success (odds ratio, 0.302; 95% confidence interval, 0.050–1.829). Only the ECOG scale was identified as an independent predictor of clinical success of SEMS placement (hazard ratio, 0.058; 95% confidence interval, 0.010–0.325). A higher ECOG scale (poor performance status) was significantly associated with failure to achieve clinical success.

**Table 3:** Multivariate analysis of factors associated with clinical success of self-expandable metallic stent placement.

		n	Odds ratio	95% CI	*P* value
Age (yrs)	<65	81			
	≥65	74	5.709	0.982–33.187	0.052
Gender	Male	102			
	Female	53	0.295	0.059–1.482	0.138
Obstruction site	Antrum/pyloric ring	96			
	Duodenal bulb/second portion	45	1.023	0.167–6.280	0.980
	Duodenal third/fourth portion	14	1.223	0.096–15.673	0.877
ECOG scale	1 or 2	119			
	3 or 4	36	0.058	0.010–0.325	0.001
Carcinomatosis	No	88			
	Yes	67	0.302	0.050–1.829	0.192

SEMS, self-expandable metallic stent; CI, confidence interval; ECOG, Eastern Cooperative Oncology Group

### Analysis of factors associated with obstructive symptom-free survival

**Fig 1:**
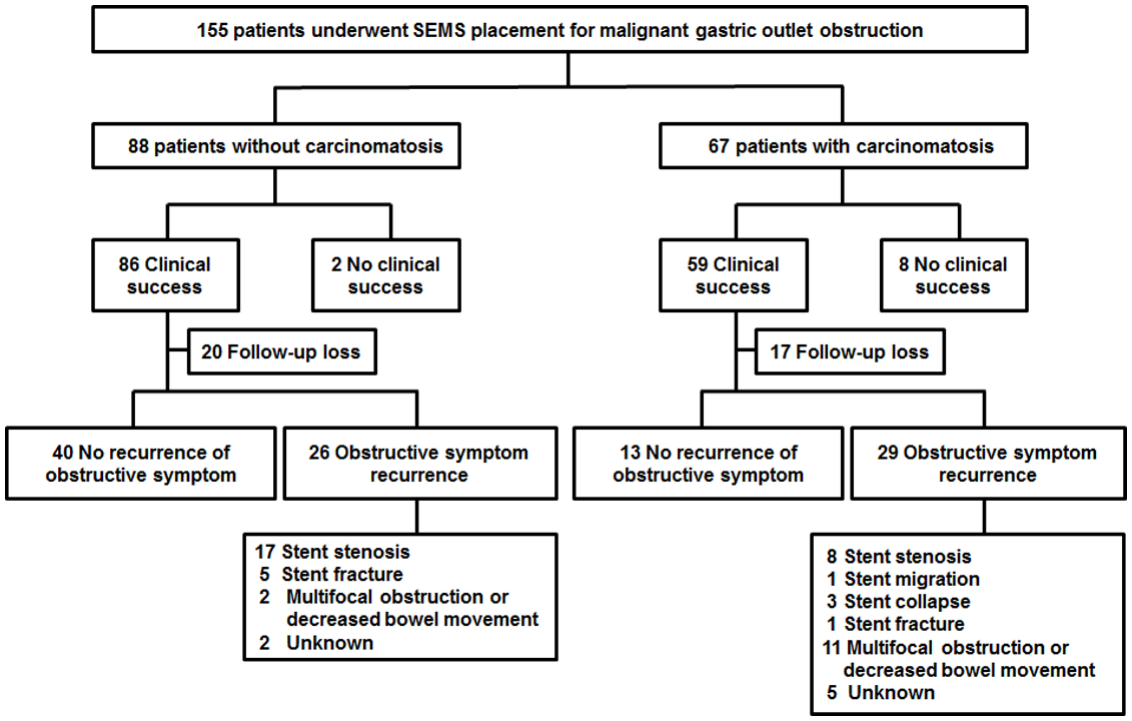
Flow chart of patients.

Fig 1 depicts a flow chart of 155 patients undergoing SEMS placement for malignant GOO. The stent patency and recurrence of obstructive symptoms was assessed in patients who had clinical success of SEMS placement and underwent follow-up (66 patients in the no carcinomatosis group; 42 patients in the carcinomatosis group). The recurrence of obstructive symptoms was observed in 39.4% (26/66) of patients in the no carcinomatosis group and 69.0% (29/42) of patients in the carcinomatosis group, respectively. In the no carcinomatosis group, stent failure was the major cause of the recurrence of obstructive symptoms accounting for 84.6% (22/26) of cases. In the carcinomatosis group, stent failure accounted for 44.8% (13/29) of cases with obstructive symptom recurrence and multifocal GI tract obstruction or decreased bowel movement accounted for 37.9% (11/29) of cases. There was a significant difference in the causes of obstructive symptom recurrence between patients with and without carcinomatosis (*P* = 0.002). The rates of receiving chemotherapy or radiation therapy after SEMS placement were 45.5% (30/66) in the no carcinomatosis group and 38.1% (16/42) in the carcinomatosis group; these rates were comparable between groups.

**Fig 2:**
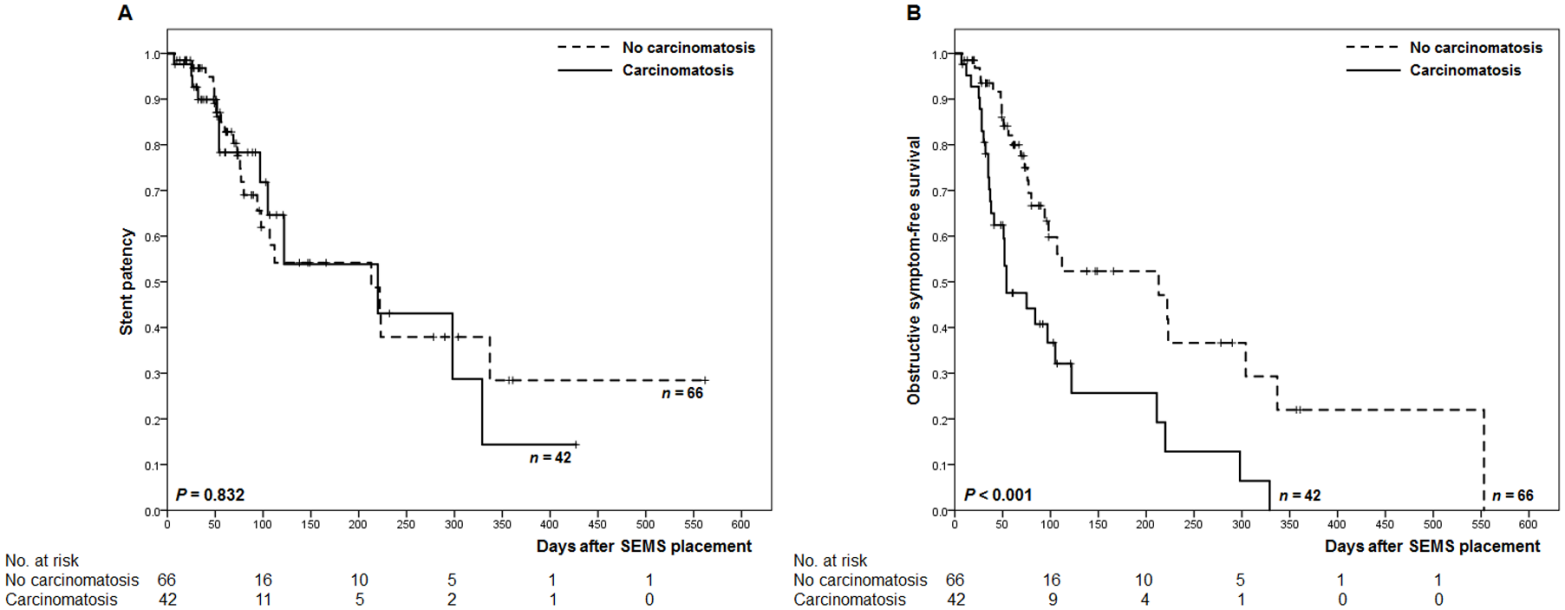
Kaplan-Meier curve of stent patency and obstructive symptom-free survival according to the presence of carcinomatosis. (A) stent patency. (B) obstructive symptom-free survival.

Fig 2 shows a Kaplan-Meier curve of stent patency and obstructive symptom-free survival according to the presence of carcinomatosis. The carcinomatosis group had comparable stent patency to the no carcinomatosis group. Stent patency rates in two months after SEMS placement were 78% in the carcinomatosis group and 83% in the no carcinomatosis group, respectively (Fig 2A). However, the carcinomatosis group showed significantly shorter obstructive symptom-free survival than the no carcinomatosis group. Obstructive symptom-free survival rates in two months after SEMS placement were 48% in the carcinomatosis group and 80% in the no carcinomatosis group, respectively (Fig 2B). In the Cox proportional-hazards model (Table 4), the presence of carcinomatosis (hazard ratio, 2.228; 95% confidence interval, 1.246–3.985), chemotherapy or radiation therapy after SEMS placement (hazard ratio, 0.480; 95% confidence interval, 0.243–0.946), and obstruction of the duodenal bulb or duodenal second portion (hazard ratio, 0.388; 95% confidence interval, 0.172–0.875) were identified as independent predictors of the obstructive symptom-free survival in patients undergoing SEMS placement for malignant GOO.

**Table 4:** Cox proportional-hazards model of obstructive symptom-free survival after self-expandable metallic stent placement.

		n	Hazard ratio	95% CI	*P* value
Age (yrs)	<65	62			
	≥65	46	0.821	0.431–1.565	0.549
Gender	Male	74			
	Female	34	1.008	0.513–1.978	0.983
Obstruction site	Antrum/pyloric ring	67			
	Duodenal bulb/second portion	31	0.388	0.172–0.875	0.022
	Duodenal third/fourth portion	10	0.519	0.193–1.394	0.193
Type of SEMS	Uncovered	96			
	Partially covered	12	0.904	0.390–2.095	0.814
Treatment after SEMS placement	No	62			
	Yes	46	0.480	0.243–0.946	0.034
ECOG scale	1 or 2	90			
	3 or 4	18	1.460	0.639–3.339	0.369
Carcinomatosis	No	66			
	Yes	42	2.228	1.246–3.985	0.007

SEMS, self-expandable metallic stent; CI, confidence interval; ECOG, Eastern Cooperative Oncology Group

## Discussion

Mendelsohn et al. [6] reported that clinical success rates immediately after SEMS placement for malignant GOO were comparable between patients with and without carcinomatosis (81% and 84%, respectively). However, two Korean studies from the same group reported that patients with carcinomatosis showed significantly lower clinical success rate than those without carcinomatosis (80.8% versus 93.9% in study by Jeon et al. [7]; 74.9% versus 90.1% in study by Park et al. [8]). Above-mentioned studies also assessed the long-term stent patency after SEMS placement for malignant GOO. Based on stent failure rate only, Mendelsohn et al. [6] and Jeon et al. [7] argued that long-term clinical outcomes after SEMS placement were comparable between patients with and without carcinomatosis. Park et al. [8] also reported that patients who had carcinomatosis but not ascites showed comparable long-term clinical outcomes to patients without carcinomatosis. However, Park et al. [8] showed that patients with both carcinomatosis and ascites had worse clinical outcomes than patients without carcinomatosis (hazard ratio, 1.4; 95% confidence interval, 1.0–1.9). Previous studies investigated the occurrence of stent failure only and did not assess the development of multifocal GI tract obstruction or decreased bowel movement after SEMS placement [6–8]. Therefore, none of above-mentioned study could fully evaluate the clinical outcomes after SEMS placement for malignant GOO in patients with carcinomatosis. To overcome this limitation, we assessed obstructive symptom-free survival, which was defined as the time period between SEMS placement and the recurrence of obstructive symptoms due to multifocal GI tract obstruction or decreased bowel movement as well as stent failure. We found that the presence of carcinomatosis was an independent predictor of the obstructive symptom-free survival in patients undergoing SEMS placement for malignant GOO.

In the present study and previous Korean studies [7, 8], patients with carcinomatosis showed significantly lower clinical success rate than those without carcinomatosis in univariate analysis. In Korean studies, the most common underlying malignancy was gastric cancer, and the most common site of obstruction was the antrum or pyloric ring. In another study by Mendelsohn et al. including a Western population [6], however, the most common underlying malignancy was pancreatic cancer, and the most common site of obstruction was the duodenal bulb or duodenal second portion. These differences in etiology and obstruction site might explain why clinical success of SEMS placement differs between Korean and Western studies [10].

In the present study, carcinomatosis was not an independent predictor of clinical success. Only the ECOG scale was identified as an independent predictor of clinical success of SEMS placement in multivariate analysis. Patients with an ECOG scale of 3 or 4 are confined to a bed or a chair for more than 50% of their waking hours, and thus, have very limited physical activity. This limited activity could result in decreased bowel movement [11, 12] and might lead to reduced clinical success rates despite successful deployment of the SEMS in the proper position across the stricture site. Carcinomatosis can lead to multifocal GI tract obstructions and may affect clinical success rates. In the present study, however, we excluded patients with radiologic evidence of multifocal GI tract obstructions before SEMS placement. Therefore, the negative effect of carcinomatosis on oral intake could have been limited at the time when clinical success was assessed (three days after SEMS placement). Given these factors, a significant association between carcinomatosis and failure to achieve clinical success shown in univariate analysis might be confounded by the strong association between the presence of carcinomatosis and the higher ECOG scale (poor performance status) (Table 1).

In contrast to short-term clinical success, the presence of carcinomatosis was identified as an independent predictor of the obstructive symptom-free survival. The recurrence of obstructive symptoms was observed in 39.4% of patients without carcinomatosis and 69.0% of patients with carcinomatosis, respectively. Multifocal GI tract obstruction or decreased bowel movement was one of the major causes of the recurrence of obstructive symptom in patients with carcinomatosis. These results supported the theoretical risk of unfavorable clinical outcomes in patients with peritoneal carcinomatosis undergoing SEMS placement for malignant GOO [1, 2, 5]. Multivariate analysis showed that chemotherapy or radiation therapy after SEMS placement was an independent predictor of the obstructive symptom-free survival. Therefore, palliative therapy after SEMS placement should be considered for patients undergoing SEMS placement for malignant GOO if these patients can tolerate additional treatment.

The present study had several limitations. First, this study was performed at a single tertiary referral center and had a retrospective design. Second, since gastric cancer was the major underlying malignancy among study population, the results might not be applicable to Western countries where pancreatic cancer is the most common underlying etiology of malignant GOO.

The results of present study showed that carcinomatosis predicts unfavorable long-term clinical outcomes in patients undergoing SEMS placement for malignant GOO, which is mainly due to the development of multifocal GI tract obstructions or decreased bowel movement as well as stent failure.
